# Association of angiogenic factors with prognosis in esophageal cancer

**DOI:** 10.1186/s12885-015-1120-5

**Published:** 2015-03-13

**Authors:** Lena Dreikhausen, Susanne Blank, Leila Sisic, Ulrike Heger, Wilko Weichert, Dirk Jäger, Thomas Bruckner, Natalia Giese, Lars Grenacher, Christine Falk, Katja Ott, Thomas Schmidt

**Affiliations:** 1 Department of General, Visceral and Transplantation Surgery, University of Heidelberg, Im Neuenheimer Feld 110, 69120 Heidelberg, Germany; 2 Department of Pathology, University of Heidelberg, 69120 Heidelberg, Germany; 3 National Center of Tumor Diseases, University of Heidelberg, 69120 Heidelberg, Germany; 4 Institute of Medical Biometry, University of Heidelberg, Heidelberg, Germany; 5 Department of Diagnostic and Interventional Radiology, University Hospital Heidelberg, Heidelberg, Germany; 6 Institute for Transplant Immunology, Hannover Medical School, Hannover, Germany

**Keywords:** Esophageal cancer, Prognosis, Angiogenic factors, Response

## Abstract

**Background:**

Despite multimodal therapy esophageal cancer often presents with poor prognosis. To improve outcome, tumor angiogenesis and anti-angiogenic therapeutic agents have recently gained importance. However, patient subgroups who benefit from anti-angiogenic therapy are not yet defined. In this retrospective exploratory study we investigated 9 angiogenic factors in patients’ serum and tissue samples with regard to their association with clinicopathological parameters, prognosis and response in patients with locally advanced preoperatively treated esophageal cancer.

**Methods:**

From 2007 to 2012 preoperative serum and corresponding tumor tissue (n = 54), only serum (n = 20) or only tumor tissue (n = 4) were collected from esophageal squamous cell carcinoma (SCC) (n = 34) and adenocarcinoma of the esophagogastric junction (AEG) (n = 44) staged cT3/4NanyM0/x after preoperative chemo(radio)therapy. Angiogenic cytokine levels in both tissue and serum were measured by multiplex immunoassay.

**Results:**

Median survival in all patients was 28.49 months. No significant difference was found in survival between SCC and AEG (p = 0.90). 26 patients were histopathological responders. Histopathological response was associated with prognosis (p = 0.05).

Angiogenic factors were associated with the following clinicopathological factors: tumor tissue expression of Angiopoietin-2 and Follistatin was higher in SCC compared to AEG (p = 0.022 and p = 0.001).

High HGF and Follistatin expression in the tumor tissue was associated with poor prognosis in all patients (p = 0.037 and p = 0.036). No association with prognosis was found in the patients’ serum. Neither patients’ serum nor tumor tissue showed an association between angiogenic factors and response to neoadjuvant therapy.

**Conclusion:**

Two angiogenic factors (HGF and Follistatin) in posttherapeutic tumor tissue are associated with prognosis in esophageal cancer patients. Biological differences of AEG and SCC with respect to angiogenesis were evident by the different expression of 2 angiogenic factors. Results are promising and should be pursued prospectively, optimally sequentially pre- and posttherapeutically.

**Electronic supplementary material:**

The online version of this article (doi:10.1186/s12885-015-1120-5) contains supplementary material, which is available to authorized users.

## Background

Esophageal cancer is known for its aggressive tumor growth and poor prognosis. 5-year overall survival rates vary between 15-25% [1]. This poor outcome is linked to the fact that the disease is often detected in an advanced state when dysphagia occurs, often making a cure by surgical resection difficult [2,3]. To improve this fatal situation, patients with locoregional disease receive neoadjuvant chemo- or chemoradiotherapy before undergoing surgery, which has been shown to provide a survival benefit [4-6]. Considering the fact that only patients who respond to this neoadjuvant therapy have a clear survival advantage, and that nonresponding patients do not, the prediction of response and prognosis is of highest interest [7-9]. Response rates differ depending on the chosen therapeutic regimen from 20–50% [7]. Even though different prediction algorithms exist, they are not yet used in clinical practice. Recent studies indicated that factors that shape the tumor microenvironment might influence patients’ response and outcome [10]. Tumor angiogenesis, the formation of new blood vessels within a tumor presents an important part of the tumor microenvironment. Beyond a certain size, tumors are not further supported by diffusion, but undergo an “angiogenic switch”, which supports further tumor growth and metastasis [11-13]. Angiogenesis within solid tumors is a complex process, involving many different factors that are active at different time points [14]. The significance of the resulting proangiogenic environment has led to the development of anti-angiogenic therapeutic agents against the main angiogenic factors. As first anti-angiogenic therapy an antibody, bevacizumab, was developed targeting against the prototypic angiogenic molecule vascular endothelial growth factor (VEGF-A). Addition of bevacizumab in metastatic colorectal cancers showed a survival benefit when added to Irinotecan and 5-Fluoruracile in a Phase III trial [15]. In Phase II studies the use of bevacizumab in patients with esophageal carcinoma in combination with Cisplatin and Capecitabine was associated with a higher response rate and longer progression-free survival. However, these trials failed to show a benefit of overall survival in patients with esophageal cancer [16]. In the REGARD trial it was recently shown that treatment with ramicirumab monotherapy, an antibody against VEGF receptor 2 (VEGFR-2), can prolong survival in patients with advanced gastric and esophageal cancer.

As a crucial element in esophageal cancer angiogenesis is linked to tumor growth and metastasis [17]. Angiogenic factors previously described in esophageal cancer are Vascular endothelial growth factor (VEGF), hepatocyte growth factor (HGF), fibroblast growth factor (FGF), midkine and thymidine phosphorylase [17]. Prognostic impact has been reported for VEGF, FGF and HGF. A recent meta-analysis on data of 29 studies and 2345 patients reexamined the role of VEGF in esophageal cancer. Esophageal SCC patients with elevated levels had a 1.82-fold greater risk of death, but no association with poor survival in AEG was shown [18]. Several studies indicate that HGF is overexpressed in SCC tissue specimen and serum levels are associated with survival and clinicopathological parameters such as distant metastases [19-21]. Another angiogenic factor associated with poor survival is FGF [22,23].

The aim of this retrospective exploratory study was to investigate the complex angiogenic cytokine expression both in tissue and serum at the time of resection in patients with neoadjuvantly treated esophageal cancer. To elucidate the complexity of this network a commercial multiplex assay for 9 angiogenic factors was utilized. Different cytokine profiles linked to therapeutic outcome could help establish anti-angiogenic targets that combined with chemo(radio)therapy could improve response to therapy in a defined subgroup of patients.

## Methods

### Patient data

78 patients with esophageal squamous cell carcinoma (n = 34) and adenocarcinoma of the gastroesophageal junction (AEG I/II) (n = 44) were included in the study. These patients were treated in the Department of Surgery, University of Heidelberg, Germany between 2007 and 2012. All patients underwent neoadjuvant chemo(radio)therapy followed by resection or explorative operation according to the current guidelines. Patients received neoadjuvant therapy as mostly on an out-patient basis from their oncologist. Therapies regimens were decided by the oncologist or after presentation at an interdisciplinary tumor board.

Patients with adenocarcinoma (44 pts) of the gastroesophageal junction were treated with either Epirubicin containing regimens (EOX, ECF) (29/44; 65,9%) or alternatively with a 5-FU/platinum based regime (FLOT/FLO/FOLFOX/PLF) (9/44; 20,5%). 5 patients (5/44; 11,4%) with AEG received chemoradiotherapy, 1 patient received a Taxol based chemotherapy. Chemoradiotherapy combined with Cisplatin and 5-Fluoruracil was given to all patients (34 pts) with esophageal squamous cell carcinoma (34/34; 100%). Details on neoadjuvant treatment regimens are shown in Additional file 1: Table S1A.

As preoperative staging all patients received a CT scan and an endoscopy. Patients with a decrease of tumor mass in endoscopy and a >50% decline in wall thickness seen in the CT and/or endoscopic ultrasound were defined as clinical responders [24].

Most patients underwent abdominothoracic en bloc esophagectomy including a 2-field-lymphadenectomy with anastomosis either in the anterior or in the posterior mediastinum or a transhiatal esophagectomy with anastomosis in the neck. Patients with AEG II received a transhiatal gastrectomy with D2-lymphadenectomy. Operative procedures are shown in Additional file 1: Table S1B. 14 Patients received adjuvant therapy. 12 received adjuvant chemotherapy, 1 additive chemotherapy and 1 palliative chemoradiotherapy.

All patients gave written informed consent. The study protocol was approved by the Ethical Committee of the University of Heidelberg.

### Follow up

Most patients received follow-up examinations in the National Center for Tumor Diseases, Heidelberg. Patient follow-up was performed quarterly in the first year, every six months in the second and third year and annually in the fourth or fifth year after resection. In case of follow-up by an oncologist, patients were contacted by phone and the oncologist was contacted by mail. Median follow-up of the surviving patients was 40.62 months, one patient was lost to follow-up.

### Histopathology

Pathological specimens were analysed in the Department of Pathology of the University Hospital Heidelberg. Histopathological staging comprised TNM classification, R-category and tumor regression rate (TRG). All patients were re-classified according to the 7^th^ edition of the TNM staging.

To specify TRG Becker regression score was used:

1a—complete regression (no residual tumor)

1b—subtotal regression (<10% residual tumor)

2—minor regression (10–50% residual tumor)

3—no regression (>50% residual tumor).

We classified patients with regression grades 1a and 1b as histopathological responders, those with grades 2 and 3 as non-responders. In addition we used 50% residual tumor as another cut-off point for response, to take the limited number of patients into account.

### Blood and tissue sampling and preparation

One day before tumor resection blood was collected in serum tubes from a central venous catheter or by peripheral venous sampling. Before blood was taken from the central venous catheter the first 5 ml of the drawn blood were discarded to avoid dilution with blocking saline. To extract the serum the tubes were centrifugated at 2.500 g for 10 minutes. Serum was then stored at −80°C until analysis. Before analysis serum was diluted 1:4 with a sample diluent.

The tissue specimens were collected directly after tumor resection and then stored at −80°C. Sections of 10 μm were cut with a cryotome. Sections were then transferred into a lysis buffer. The concentration of the lysed tissue samples was adjusted to 600 μg/ml.

### Cytokine detection

We detected serum and tissue concentrations of Platelet Endothelial Cell Adhesion Molecule 1 (PECAM-1), Vascular Endothelial Growth Factor (VEGF), Leptin, Angiopoietin-2 (Ang-2), Follistatin, Granulocyte-Colony Stimulating Factor (GCSF), Hepatocyte Growth Factor (HGF), Platelet-Derived Growth Factor (PDGF) and Interleukin-8 (IL-8). Cytokine levels were measured using the BioRad Bio-Plex Human Angiogenesis Assay (Bio-Rad Laboratories, Inc., Hercules, CA 94547, USA) and Luminex two-laser array reader (Bioplex200). Bioplex Manager 6.1. (Bio-Rad Laboratories, Inc., Hercules, CA 94547, USA) was used to acquire standard curves and concentrations.

### Statistical analysis

Continuous variables are expressed as median and inter quartile ranges (IQR) with additional 95% confidence interval. To compare differences in medians we used the Mann–Whitney-U test or Kruskal-Wallis H-Test for multiple comparisons between the groups. Additional multiple ANOVA (MANOVA) testing was performed for multiple hypothesis testing in multiple comparisons. Categorical data is presented in absolute and relative frequencies. Comparison was performed by the Chi-square-test. To compare angiogenic cytokine levels we used the median as a cut-off.

We measured overall survival from point of diagnosis until death. The Kaplan Meier Method was performed for survival analysis, for differences in survival time we used the log-rank test. Results for overall survival were confirmed with multi-variate Cox regression analysis. Receiver-operating characteristic (ROC) curves and area under the ROC curve (AUC) were used to assess the diagnostic value of Follistatin and HGF. A ROC curve was created to select the Youden’s index (Youden’s index = sensitivity + specificity − 1), and the highest sensitivity and specificity were selected as the cutoff values. Statistical significance was taken as a p-value of <0.05 (two-tailed). All statistical analyses were done using SPSS software version 20.0 (SPSS, Inc., Chicago, Illinois, USA).

## Results

Patients’ pretherapeutic and postoperative clinicopathological characteristics are displayed in Tables 1 and 2. We included 78 patients, 34 (43.6%) with esophageal squamous cell carcinoma and 44 (56.4%) with adenocarcinoma of the gastroesophageal junction (AEG I/II).

**Table 1:** **Pretherapeutic patients’ characteristics**

Characteristics		n	%	Median survival (IQR)	95%CI	3-Y-S (%)	p value
Total, n		78	100,0%	28,5 (13,7;*)		42,7%	
Age (years)	63 ± 7,8 (41–80)						
Sex	Male	62	79,5%	28,5 (13,8;*)	16,1 - 40,9	42,6%	0,711
	Female	16	20,5%	29,1 (11,6;*)	-	45,6%	
Localisation	AEG I/II	44	56,4%	28,5 (17,0;*)	19,2 - 37,8	37,7%	0,904
	SCC	34	43,6%	n.r.	-	50,9%	
cT Category	cT1	0					
	cT2	5	6,4%	n.r.	-	60,0%	0,812
	cT3	69	88,5%	23,8 (12,8;*)	15,9 - 31,7	42,8%	
	cT4	4	5,1%	36,7 (33,7;*)	31,9 - 41,5	33,3%	
cN Category	cN0	17	21,8%	30,6 (20,5;*)	15,5 - 45,7	37,8%	0,906
	cN+	61	78,2%	24,0 (12,3;*)	9,1 - 38,8	44,6%	
cM Category	cM0	67	85,9%	23,8 (12,8;*)	15,1 - 32,5	40,2%	0,317
	cM1	11	14,1%	n.r.	-	57,3%	
Grading	G 1/2	32	41,0%	23,1 (11,0;*)	13,4 - 32,7	34,0%	0,428
	G 3/4	38	48,7%	29,1 (13,8;*)	-	45,5%	
Lauren initial (AEG)	Intestinal	26	59,1%	30,6 (17,0;*)	15,6 - 45,6	40,4%	0,620
	Nonintestinal	13	29,5%	24,0 (20,3;*)	19,3 - 28,6	33,8%	

Median Survival shown in months; n.r.: not reached; CI: confidence interval; 3-Y-S: 3-Year-Survival; IQR: inter quartile range (1^st^ quartile; 3^rd^ quartile (* 3^rd^ quartile not reached)).

**Table 2:** **Postoperative patients’ characteristics**

Characteristics		n	%	Median Survival (IQR)	95%CI	3-Y-S (%)	p Value
pT Category	pT0	17	21,8%	n.r.	-	54,1%	0,464
	pT1	8	10,3%	n.r.	-	72,9%	
	pT2	10	12,8%	n.r.	-	58,3%	
	pT3	38	48,7%	23,8 (14,0;*)	14,3 - 33,4	33,8%	
	pT4	4	5,1%	10,4 (3,9;19,1)	0,0 - 25,2	25,0%	
pN Category	pN0	36	46,2%	n.r.	-	66,1%	**0,001**
	pN+	40	51,3%	20,3 (11,9;33,7)	14,9 - 25,6	21,8%	
pM Category	pM0	74	94,9%	n.r.	-	46,1%	0,235
	pM1	3	3,8%	21,8 (3,9;33,7)	0,0 - 73,3	0,0%	
Grading postop.	G1/2	15	19,2%	22,6 (11,6;*)	-	48,1%	0,927
	G3/4	33	42,3%	28,5 (13,7;*)	15,5 - 41,4	40,7%	
Lauren postop. (AEG)	Intestinal	22	50,0%	24,0 (19,1;*)	14,6 - 33,3	27,8%	0,796
	Nonintestinal	14	31,8%	21,1 (13,8;*)	15,7 - 26,6	31,2%	
R Status	R0	68	87,2%	29,1 (13,8;*)	15,8 - 42,4	42,7%	0,484
	R1/2	8	10,2%	13,7 (10,4;*)	0,0 - 27,4	37,5%	

Median Survival shown in months; n.r.: not reached; CI: confidence interval; 3-Y-S: 3-Year-Survival; IQR: inter quartile range (1^st^ quartile; 3^rd^ quartile (* 3^rd^ quartile not reached)).

### Prognostic clinicopathological factors

As relevant prognostic factors in all patients we found the pN-category (p = 0.001). Additionally tumor regression grade (TRG) was found to be a significant prognostic factor in all patients (p = 0.052) and in adenocarcinoma of the gastroesophageal junction (p = 0.048). Clinical and histopathological reponse and 3-year survival rates of SCC and AEG are displayed in Additional file 2: Table S2.

### Correlation of angiogenic factors with clinicopathological factors

Angiopoietin-2 and Follistatin protein levels within the tumor tissue were associated with tumor type (p = 0.022 and p = 0.001). Patients with SCC had higher levels of Angiopoietin-2 and Follistatin levels than patients with AEG (Table 3). Circulating angiogenic factors did not correlate with other clinicopathological factors (Table 4).

**Table 3:** **Correlation of angiogenic factors of the tumor tissue and clinicopathological factors**

Characteristics		G-CSF	PECAM-1	HGF	VEGF	Leptin	PDGF-BB	Ang-2	Foll.	IL8	Ang2/VEGF
Gender	male	6,1	34366,0*	5451,8	39,2	105,5	37,1*	355,0	552,7	59,7	5,7
	female	4,2	28126,3*	4166,9	59,0	127,2	26,4*	392,2	712,8	62,9	4,3
Localisation	AEG I/II	6,1	27041,8	4848,4	39,2	108,2	33,3	316,1*	408,5*	59,7*	4,7
	SCC	8,0	31757,8	5618,5	59,0	127,2	29,1	464,7*	820,1*	59,7*	11,4
Grading	G1/2	4,5	32785,4	4042,4	39,2	105,5	35,6	363,2	624,3	37,6	6,2
	G3/4	6,6	28933,8	5451,8	49,8	124,5	30,6	379,8	560,4	62,9	5,0
cT Category	cT1										
	cT2	16,4	33440,3	1969,7	80,5	205,3	97,5	105,2	705,9	179,7	1,3
	cT3	6,1	29392,7	5394,0	38,1	105,5	33,3	375,1	597,3	39,0	7,6
	cT4	6,1	34773,5	11315,6	184,8	111,0	30,2	384,7	342,4	116,6	3,4
cN Category	cN0	6,1	34933,9	5440,2	41,8	116,3	29,1	396,4	624,3	38,4	8,5
	cN+	6,1	30954,5	5239,3	47,8	108,2	33,3	353,8	552,7	59,7	5,0
cM Category	cM0	6,4	29583,5	5316,6	40,0	113,6	32,9	371,5	578,9	49,4	6,1
	cM1	5,9	36863,4	6869,7	61,8	101,4	32,5	358,1	529,7	66,0	1,2
pT Category	pT0	4,7	30954,5	4042,4	24,9	89,4	26,8	392,8	712,8	20,4	17,1
	pT1	11,0	38189,0	5248,9	55,0	195,0	62,0	223,5	538,1	152,1	4,1
	pT2	5,3	30783,3	7546,9	51,6	101,4	37,5	312,6	751,5	119,7	3,8
	pT3	6,8	32469,4	6338,5	56,4	116,3	36,2	386,0	486,4	66,0	4,8
	pT4	17,3	20880,8	732,2	108,1	108,2	48,7	396,4	101,4	821,3	0,7
pN Category	pN0	8,0	33440,3	4848,4	41,8	119,0	33,3	424,8	624,3	59,7	10,1
	pN+	6,1	28126,3	5618,5	43,2	108,2	31,7	353,8	487,5	59,7	4,7
pM Category	pM0	6,4	30364,4	5417,1	42,5	109,5	31,9	365,6	557,7	59,7	5,7
	pM1	5,4	35429,1	8653,0	161,6	113,7	64,6	456,4	584,1	40,1	19,1
Response											
Clinical	Resp.	4,7	33440,3	4042,4	41,8	127,2	28,7	424,8	712,8*	37,5	11,4*
	Nonresp.	6,6	28709,3	5968,0	45,5	106,8	36,2	365,6	557,7*	61,3	4,9*
Histopathological	Resp.	4,7	29774,3	4848,4	25,9	94,7	28,7	392,8	731,1	21,8	11,4*
	Nonresp.	6,6	32785,4	6317,4	51,1	116,3	37,1	363,2	487,5	66,2	4,7*

Values are the median values of the particular subgroups in pg/ml; statistically significant factors identified by the Mann–Whitney-U-Test/Kruskal-Wallis-Test are marked in bold; (significant factors identified by MANOVA are marked with*)Ang-2: Angiopoietin-2; Ang2/VEGF: ratio between Angiopoietin-2 and VEGF.

**Table 4:** **Correlation of circulating angiogenic factors and clinicopathological factors**

Characteristics		G-CSF	PECAM-1	HGF	VEGF	Leptin	PDGF-BB	Ang-2	Foll.	IL8	Ang2/VEGF
Gender	male	35,7*	3278,0	1016,1	38,4	1468,8	1381,3	869,3	200,4	11,1	21,9
	female	35,3*	3052,5	965,2	36,5	2083,6	961,4	802,9	209,1	12,2	21,2
Localisation	AEG I/II	35,7	3295,3	1023,4	37,1	1624,9	1185,4	941,6	213,9	12,3	22,3
	SCC	36,7	3017,6	921,7	37,1	1079,8	1334,8	776,1	192,7	11,0	20,8
Grading	G1/2	37,6	3415,3	950,7	37,1	1474,8	1621,6	869,3	242,9*	12,7	22,1
	G3/4	34,8	3260,8	1023,4	38,8	1733,7	1032,3	820,3	196,5*	11,6	22,2
cT Category	cT1										
	cT2	28,3	3364,0*	763,5	38,1	2250,5	1082,1	757,5	188,8	9,0	19,9
	cT3	35,7	3156,8*	979,7	36,1	1468,8	1242,4	867,0	199,4	11,3	22,4
	cT4	54,4	6421,4*	1318,4	212,2	1114,8	4365,2	1331,2	393,7	14,4	6,6
cN Category	cN0	34,8	3260,8	900,1	37,4	1349,8	1088,7	1212,8*	194,6	10,9	20,1
	cN+	35,7	3226,3	1019,8	37,4	1636,9	1387,2	832,0*	205,2	12,3	22,4
cM Category	cM0	35,7	3295,3	965,2	38,8	1624,9	1250,7	891,5	215,8	11,6	22,4
	cM1	37,6	2663,4	1170,2	29,3	1255,3	1334,8	685,3	183,1	12,3	19,6
pT Category	pT0	28,3	2841,5	849,5	38,8	1103,1	1500,6	757,5	221,6	10,9	14,1
	pT1	43,7	3260,8	997,9	29,3	2908,3	1175,4	722,6	194,6	10,0	21,6
	pT2	37,6	2717,0	1694,2	37,4	2501,2	1418,6	1021,2	201,3	14,1	18,2
	pT3	35,7	3398,2	1023,5	33,5	1314,4	1137,1	868,1	210,0	11,3	23,4
	pT4	27,3	4136,8	950,7	62,7	3011,1	1427,7	583,1	146,8	9,5	14,7
pN Category	pN0	35,7	3226,3	1023,4	50,4	1456,9	1499,8	941,6	204,2	11,0	18,7
	pN+	35,3	3278,0	961,6	32,4	1552,9	1180,4	818,0	210,0	12,2	22,9
pM Category	pM0	35,7	3226,3	979,7	36,5	1492,7	1275,1	867,0	209,0	11,3	21,9
	pM1	41,3	4136,8	950,7	39,4	1114,8	1088,7	778,4	171,6	12,3	14,7
Response											
Clinical	Resp.	28,3	3226,3	961,6	44,1	1880,0	1500,6	945,1	194,6	12,3	22,7
	Nonresp.	35,7	3295,3	1023,4	36,5	1361,7	1250,7	843,6	204,2	11,6	21,4
Histopathological	Resp.	34,3	3017,6	921,7	37,8	1733,7	1500,6	727,2	194,6	11,0	18,1
	Nonresp.	35,7	3278,0	1045,4	35,1	1468,8	1225,6	891,5	209,0	12,2	22,4

Values are the median values of the particular subgroups in pg/ml; significant factors identified by MANOVA are marked with *; (no statistically significant factors were identified by the Mann–Whitney-U-Test/Kruskal-Wallis-Test); Ang-2: Angiopoietin-2; Ang2/VEGF: ratio between Angiopoietin-2 and VEGF.

### Angiogenic cytokines and response to chemotherapy

As shown in Tables 3 and 4 angiogenic cytokine levels either circulating or within the tissue were not associated with clinical response or TRG for all patients as well as in the AEG and SCC subgroups (Additional file 3: Table S3). Defining histopathological non-responding patients as having more than 50% remaining tumor cell mass in histopathological analysis, histopathological non-responders showed lower tissue levels of Follistatin than patients with more than 50% remaining tumor cells (p = 0.015).

### Prognostic impact of angiogenic cytokines

**Figure 1:**
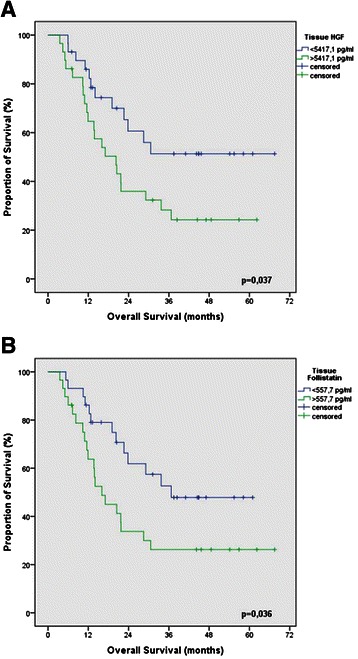
**Prognostic cytokines in the tissue of esophageal cancer patients.** Kaplan-Maier plots for overall survival of esophageal cancer patients according to **A)** tissue protein levels of HGF (smaller and higher then median of 5417,1 pg/ml) and **B)** tissue protein levels of follistatin (smaller and higher then median of 557,7 pg/ml) (n = 58; p < 0.05).

To evaluate the prognostic impact of the angiogenic cytokines, Kaplan-Maier survival analysis were performed for each angiogenic factor with the median as cut-off. In the patients serum no angiogenic cytokine was found to be associated with survival time. In the tissue samples HGF and Follistatin levels were associated with patients’ prognosis (p = 0.037; p = 0.036) (Figures 1A and B). Median survival of patients with lower levels of HGF was not reached at time of analysis, while median survival of patients with higher levels was 20.3 ± 4,3 (11,7 – 28,8 95%CI) months. Median survival of patients with lower levels of Follistatin was 36,7 months (95% CI not reached), compared to 16,0 ± 2,7 (10,6 – 21,4 95% CI) months for patients with higher levels. The median survival and the significant cytokines for all patients are presented in Tables 5 and 6. When performing multivariate cox regression analysis including all angiogenic factors in tissue, Follitstatin levels remained associated to overall survival (p = 0.04).

**Table 5:** **Prognostic value of angiogenic factors in the tumor tissue**

Tissue factor	Median		n	%	Median survival (IQR)	95% CI	3-Y-S (%)	p Value
G-CSF	6,1	≤ Median	30	51,7%	33,7 (17,0;*)	15,7 - 51,8	40,6%	0,181
		> Median	28	48,3%	14,0 (10,5;*)	2,5 - 25,6	32,1%	
PECAM-1	31356,1	< Median	29	50,0%	29,1 (12,3;*)	-	47,9%	0,251
		> Median	29	50,0%	22,6 (11,9;*)	13,9 - 31,3	28,7%	
**HGF**	**5417,1**	**< Median**	**29**	**50,0%**	**n.r.**	**-**	**51,3%**	**0,037**
		**> Median**	**29**	**50,0%**	**20,3 (10,9;36,7)**	**11,7 - 28,8**	**24,2%**	
VEGF	42,5	< Median	29	50,0%	23,8 (17,0;*)	12,9 - 34,8	36,6%	0,475
		> Median	29	50,0%	22,6 (10,5;*)	0,0 - 47,7	36,9%	
Leptin	109,5	< Median	29	50,0%	22,6 (14,0;*)	4,0 - 41,3	40,6%	0,420
		> Median	29	50,0%	21,7 (10,9;*)	4,8 - 38,7	33,1%	
PDGF-BB	32,5	< Median	29	50,0%	28,5 (14,0;*)	19,0 - 38,0	32,3%	0,777
		> Median	29	50,0%	21,7 (10,9;*)	10,4 - 32,9	40,3%	
Angiopoietin-2	371,5	< Median	29	50,0%	22,6 (11,9;*)	1,3 - 44,0	40,3%	0,712
		> Median	29	50,0%	23,8 (12,3;*)	9,7 - 37,9	34,5%	
**Follistatin**	**557,7**	**< Median**	**29**	**50,0%**	36,7 (19,1;*)	**-**	**47,9%**	**0,036**
		**> Median**	**29**	**50,0%**	16,0 (10,9;*)	**10,6 - 21,4**	**26,2%**	
IL-8	59,7	≤ Median	30	51,7%	21,7 (13,8;*)	2,8 - 40,7	41,0%	0,431
		> Median	28	48,3%	22,6 (10,5;*)	12,1 - 33,1	32,5%	
Ang-2/VEGF-Ratio	5,7	< Median	29	50,0%	33,7 (11,9;*)	-	46,0%	0,594
		> Median	29	50,0%	21,7 (13,8;*)	11,7 - 31,8	30,0%	

Median survival shown in months; n.r.: not reached; CI: confidence interval; 3-Y-S: 3-Year-Survival; IQR: inter quartile range (1^st^ quartile; 3^rd^ quartile (* 3^rd^ quartile not reached)); statistically significant factors are marked in bold.

**Table 6:** **Prognostic impact of serum angiogenic factors**

Serum factor	Median		n	%	Median survival (IQR)	95% CI	3-Y-S (%)	p value
G-CSF	35,7	≤ Median	41	55,4%	23,0 (12,8;*)	12,8 - 33,2	40,6%	p = 0,669
		> Median	32	43,2%	30,6 (16,0;*)	16,2 - 45,0	43,5%	
PECAM-1	3243,5	< Median	37	50,0%	29,1 (12,8;*)	17,1 - 41,1	44,5%	p = 0,994
		> Median	37	50,0%	28,5 (17,0;*)	12,3 - 44,7	40,8%	
HGF	979,7	≤ Median	37	50,0%	30,6 (12,8;*)	12,9 - 48,3	43,0%	p = 0,871
		> Median	36	48,6%	28,5 (13,7;*)	15,7 - 41,3	41,1%	
VEGF	37,1	< Median	36	48,6%	23,0 (12,3;*)	12,9 - 33,1	39,8%	p = 0,590
		> Median	36	48,6%	33,7 (16,0;*)	14,8 - 52,6	44,3%	
Leptin	1492,7	≤ Median	37	50,0%	30,6 (14,0;*)	16,5 - 44,7	43,5%	p = 0,800
		> Median	36	48,6%	28,5 (11,6;*)	10,0 - 47,0	41,6%	
PDGF-BB	1275,1	< Median	37	50,0%	23,8 (12,3;*)	14,3 - 33,3	39,6%	p = 0,396
		> Median	37	50,0%	36,7 (18,8;*)	-	46,2%	
Angiopoietin-2	867,0	≤ Median	37	50,0%	29,1 (16,0;*)	18,8 - 39,4	44,7%	p = 0,606
		> Median	36	48,6%	28,5 (11,6;*)	11,2 - 45,8	39,7%	
Follistatin	200,37	< Median	37	50,0%	30,6 (16,0;*)	-	47,4%	p = 0,416
		> Median	37	50,0%	23,8 (12,8;*)	7,1 - 40,5	38,3%	
IL-8	11,86	< Median	37	50,0%	n.r.	-	51,5%	p = 0,143
		> Median	37	50,0%	21,7 (12,3;*)	17,3 - 26,1	32,6%	
Ang-2/VEGF-Ratio	21,9	< Median	36	48,6%	33,7 (19,1;*)	14,9 - 52,5	44,3%	p = 0,379
		> Median	36	48,6%	23,0 (11;*)	11,6 - 34,4	39,0%	

Median survival shown in months; n.r.: not reached; CI: confidence interval; 3-Y-S: 3-Year-Survival; ; IQR: inter quartile range (1^st^ quartile; 3^rd^ quartile (* 3^rd^ quartile not reached)).

In AEG tumor tissues alone HGF and Leptin were found to be associated with patients’ survival (p = 0.028 and p = 0.034) (Additional file 4: Tables S4 A and B). In SCC alone no angiogenic cytokine in the tumor tissue reached statistical significance. A lower Angiopoietin-2/VEGF-Ratio in the serum was associated with longer survival in patients with SCC (p = 0.032) (Additional file 4: Tables S4 C and D).

### Receiver Operating Characteristics (ROC) curves

We performed Receiver Operating Characteristics for the statistically significant cytokines to determine possible cut-offs that are more appropriate than the median. The calculated optimal cut-offs are similar to the ones using the median as cut-off. Receiver operating charateristics with the optimal cut-offs, sensitivity and specificity are shown in Table 7.

**Table 7:** **Receiver Operating Characteristics (ROC) for relevant factors**

Tissue factor	Median	Sensitivity/specificity	Best cut-point	Sensitivity/specificity	AUC
HGF	5417,09	63,6/68	5316,62	66,7/68	0,697
Follistatin	557,66	60,6/64	666,22	54,5/72	0,568

Median / Best cut-point values in pg/ml; AUC: area under the curve.

## Discussion

In this study a panel of angiogenic factors in neoadjuvantly treated patients with esophageal cancer (AEGI/II and SCC) that underwent tumor resection in the Department of Surgery at the University of Heidelberg was analyzed in the tumor tissue and serum. Of the angiogenic factors VEGF, Angiopoietin-2, PECAM-1, Follistatin, Leptin, G-CSF, HGF, PDGF-BB and IL-8, two angiogenic factors (HGF and Follistatin) are associated with esophageal cancer patients’ prognosis in posttherapeutic tumor tissue. Expression levels of most angiogenic factors in AEG and SCC were similar, biological differences of these two entities of esophageal cancer with respect to angiogenesis are indicated by the different expression of Angiopoietin-2 and Follistatin. No association with response to neoadjuvant therapy could be observed.

Results of this study are of current importance as antiangiogenic therapy has found its way into clinical therapy regimens. As a result the complex interactions in the tumor microenvironment and the role of angiogenesis within it receive more and more interest. It has yet to be determined if the angiogenic microenvironment influences neoadjuvant therapy response in esophageal cancer patients. Currently reliable clinical or molecular markers to evaluate response are still lacking, even though metabolic response prediction has recently been evaluated as a method [25]. Response prediction is becoming of exceeding interest especially in regard to the chosen therapeutical regimen considering that response rates to classical chemo(radio)therapeutical regimens do mostly not exceed 50% [7]. This analysis of the angiogenic phenotype within the tumor and its tumor microenvironment and its relation to therapy response adds further insight into this problem. As key hallmark of cancer, angiogenesis is activated early in tumorigenesis and contributes to tumor progression and metastasis by influencing and shaping the tumor microenvironment [11]. The “angiogenic switch” occurs when the balance between pro- and anti-angiogenic factors is shifted towards the pro-angiogenic side, resulting in increased angiogenesis and tumor growth [26]. In esophageal cancers angiogenesis has been described to be involved in tumor growth and metastasis [17] and the prototypic angiogenic factor VEGF is often associated with poor survival. Other pro-angiogenic factors such as HGF, FGF and IL-8 have been reported to play a role in esophageal cancer [18,27]. However most studies were limited to one or two investigated factors, curbing conclusions about the complex network of different angiogenic factors. A strength of our study is the analysis of a comprehensive angiogenic factor panel in both blood and corresponding tumor tissue in most of the patients. Furthermore all included patients underwent neoadjuvant treatment.

Limitations of our study are the limited number of patients and the retrospective design, with all its known and accepted problems. Furthermore, all esophageal cancer entities are analyzed together, which might be criticized by some groups, despite the fact that the UICC classification [28] and landmark studies like the CROSS Study [5,29] also combine them. To address this issue we additionally provided subgroup analysis. As further limitations both tumor and serum samples was available in only 70% of the patients and different preoperative treatment regimens were applied, although both are accepted standards in esophageal cancer [4-6,30,31].

We found that in the tumor tissue Follistatin and Angiopoietin-2 are differentially expressed between the two tumor types with significantly higher median tissue levels of the two angiogenic factors in SCC patients when compared to AEG patients. These general differences in expression support the hypothesis that AEG and SCC represent cancer types with at least some biological differences in angiogenesis. Similarly differences in etiology, epidemiology and tumor biology between AEG and SCC have been reported before [2], but both tumor entities are classified identically within UICC 2010 [28] and analyzed together in most studies [5].

Hepatocyte Growth Factor (HGF) and Follistatin in the tumor tissue were associated with patient survival. Using the median as cut-off, high HGF and Follistatin expression in the tumor tissue were associated with poor patients’ prognosis. ROC analysis aimed to evaluate if a more appropriate cut-off value then the median should be used. However the calculated “optimal” cut-off values were actually close to the used median and support its use as cut-off value in this study. The low value of the AUC is likely related to the small sample size for this calculation, however it underlines the need of additional larger studies in the future.

HGF and its receptor c-Met play an important role in the development of various cancer types. HGF, also known as scatter factor is a growth factor directed to epithelial cells that is active in embryogenesis and mediates defense to tissue damage in adults. HGF binds c-Met, a tyrosine kinase receptor as exclusive ligand induces the activation of oncogenic pathways, angiogenesis and scattering of cells, leading to metastasis [32]. Supporting our findings concerning HGF, Tuynman et al. found Met expression to be an independent prognostic risk factor in esophageal adenocarcinoma [33]. Furthermore, it has been reported that higher HGF levels in serum and tissue are associated with tumor progression and poor survival in esophageal squamous cell carcinoma [19,20]. Ren et al. found pretherapeutic HGF levels as an independent prognostic factor [19]. In our data we did not find a correlation of HGF in the patients’ sera with survival. This could be due to the point of time when the blood sampling took place. Neoadjuvant therapy may influence HGF serum levels in a different way than in the tumor tissue.

Follistatin tissue levels were significantly associated with patients’ prognosis. Elevated levels of Follistatin as an antagonist of the TGFβ superfamily member Activin A have been reported in solid tumors [34-37], however no data on esophageal cancer have been published so far. Interestingly tissue levels of Follistatin differed between non-responders and responders, when defining responders as having less than 50% remaining tumor cell mass (p = 0.015). The definitions of histopathological response after chemotherapy or radiochemotherapy are heterogenous varying from a pCR up to 50% residual tumor and would need clear definitions to make study results comparable.

We investigated angiogenic cytokine levels with regard to patients’ clinical and histopathological response and prognosis. Considering the fact that all patients received neoadjuvant chemo(radio)therapy it is interesting that we found no association between circulating angiogenic cytokines and patients survival or response. This could be related to the point in time of posttherapeutic measurement of the factors. Neoadjuvant therapy might influence circulating angiogenic cytokine levels and thus change levels after chemo(radio)therapy. Most studies investigated angiogenic factors in previously untreated patients [19,38-41].

Based on these interesting obtained results we would recommend a prospective validation of this data in a patient cohort from multiple centers. If the results are confirmed a large multicenter trial with a central bio-banking should be performed. This should be included within the next trials on neoadjuvant or perioperative chemo(radio)therapy. It would be favorable to obtain blood samples in a standardized manner, pre-therapeutic, during therapy, before surgery and during the follow up. Tumor tissue could also be obtained during initial biopsy and from the pathological sample. With this information it will be possible to provide a more complete picture of the tumor biology. The aim will be to identify deregulated targetable pathways as i.e. the HGF/met pathway.

In summary, further studies will be necessary to investigate the impact of neoadjuvant therapy on tumor microenvironment and circulating angiogenic cytokines. Pretherapeutic and preoperative blood sampling could elucidate the change of angiogenic factor levels over time of therapy. As the limited number of patients may have influenced the results of this study, investigations in larger patient cohorts could confirm our findings and evaluate angiogenic cytokine levels as pretherapeutic diagnostic factors – helping the physician to choose the right therapy.

## Conclusion

Angiogenic cytokines seem to play an important role in patients with esophageal cancer. In this study two angiogenic factors (HGF and Follistatin) in tumor tissue after neoadjuvant therapy were associated with esophageal cancer patients’ prognosis. HGF levels seem to be important with regard to tumor development in esophageal cancer. Different expression of angiogenic cytokines in AEG and SCC subgroups confirm the assumption that the two represent different entities with respect to angiogenesis. The lack of association with response to therapy could be explained by the fact that samples were collected at a preoperative and not pretherapeutic point. Results are promising and should be pursued prospectively in a pre- and posttherapeutic state. Further studies with larger number of patients seem to be necessary.

## Additional files

Additional file 1: Table S1. A) neoadjuvant treatment regimens. B) operative procedures.
Additional file 2: Table S2. Response to neoadjuvant therapy.
Additional file 3: Table S3. Association of angiogenic cytokines with clinical response and tumor regression grade (TRG) in AC and SCC.
Additional file 4: Table S4. A) Prognostic significance of circulating angiogenic factor levels in AEG I/II. B) Prognostic significance of tissue angiogenic factor levels in AEG I/II. C) Prognostic significance of circulating angiogenic factor levels in SCC. D) Prognostic significance of tissue angiogenic factor levels in SCC.
