# Supplementary material for: Association of angiogenic factors with prognosis in esophageal cancer
Source: BMC Cancer. 2015 Mar 13;15:121. doi: 10.1186/s12885-015-1120-5 (PMC4362831; doi:10.1186/s12885-015-1120-5)
Supplement: Additional file 1: Table S1. — A) neoadjuvant treatment regimens. B) operative procedures. [file 12885_2015_1120_MOESM1_ESM.docx]

**Additional file 1: Table S1**

**A) neoadjuvant treatment regimens**

| **Regimen** | **n** | **%** |
| --- | --- | --- |
| Chemoradiotherapy | 39 | 50 |
| EOX | 26 | 33,3 |
| FLO(T) | 4 | 5,1 |
| Others | 9 | 11,5 |

EOX: epirubicin, oxaliplatinum, capecitabine;

FLO: oxaliplatinum, 5-fluorouracil, folinic acid;

FLOT: docetaxel, oxaliplatinum, 5-fluorouracil, folinic acid

i.e. EOX regimen (Sumpter K et al. Br J Cancer 2005; Cunningham D et al. NEJM 2008)

i.e. FLOT regimen (Al-Batran SE et al. Ann of Oncol 2008; Al-Batran et al. JCO 2008)

**B) operative procedures**

| **Type of Surgery** | **n** | **%** |
| --- | --- | --- |
| Abdominothoracic esophagectomy | 62 | 79,5% |
| Transhiatal extended gastrectomy | 13 | 16,7% |
| Transmediastinal esophagectomy | 2 | 2,6% |
| Exploration | 1 | 1,3% |
