# Supplementary material for: Association of angiogenic factors with prognosis in esophageal cancer
Source: BMC Cancer. 2015 Mar 13;15:121. doi: 10.1186/s12885-015-1120-5 (PMC4362831; doi:10.1186/s12885-015-1120-5)
Supplement: Additional file 2: Table S2. — Response to neoadjuvant therapy. [file 12885_2015_1120_MOESM2_ESM.docx]

**Additional file 2: Table S2: Response to neoadjuvant therapy**

| **Response** |  | **n** | **%** | **Median Survival**  **(IQR)** | **95% CI** | **3-Y-S (%)** | **p Value** |
| --- | --- | --- | --- | --- | --- | --- | --- |
| **Clinical** |  |  |  |  |  |  |  |
| All patients | Responder | 21 | 26,9% | n.r. | - | 65,2% | 0,126 |
|  | Nonresponder | 55 | 70,5% | 23,8 (13,7;*) | 14,2 - 33,4 | 34,7% |  |
| AEG I/II | Responder | 8 | 18,2% | n.r. | - | 72,9% | 0,098 |
|  | Nonresponder | 35 | 79,5% | 24,0 (13,8;*) | 15,0 - 32,8 | 29,4% |  |
| SCC | Responder | 13 | 38,2% | n.r. | - | 61,5% | 0,628 |
|  | Nonresponder | 20 | 58,8% | 20,5 (11,6;*) | - | 46,8% |  |
| **Histopathological** | |  |  |  |  |  |  |
| All patients | Responder | 26 | 33,3% | n.r. | - | 61,3% | 0,052 |
|  | Nonresponder | 51 | 65,4% | 22,6 (12,8;*) | 18,2 - 27,0 | 33,9% |  |
| AEG I/II | Responder | 7 | 15,9% | n.r. | - | 71,4% | **0,048** |
|  | Nonresponder | 37 | 84,1% | 23,8 (13,7;*) | 20,2 - 27,5 | 30,0% |  |
| SCC | Responder | 19 | 55,9% | n.r. | - | 60,6% | 0,355 |
|  | Nonresponder | 14 | 41,2% | 18,8 (10,4;*) | 7,0 - 30,6 | 42,9% |  |

**Median Survival shown in months; n.r.: not reached; CI: confidence interval; 3-Y-S: 3-Year-Survival; IQR: inter quartile range (1^st^ quartile; 3^rd^ quartile)**
