# Supplementary material for: Association of angiogenic factors with prognosis in esophageal cancer
Source: BMC Cancer. 2015 Mar 13;15:121. doi: 10.1186/s12885-015-1120-5 (PMC4362831; doi:10.1186/s12885-015-1120-5)
Supplement: Additional file 3: Table S3. — Association of angiogenic cytokines with clinical response and tumor regression grade (TRG) in AC and SCC. [file 12885_2015_1120_MOESM3_ESM.docx]

**Additional file 3: Table S3: Association of angiogenic cytokines with Clinical Response and TRG in AC and SCC**

| **AEG I/II** |  | **G-CSF** | **PECAM-1** | **HGF** | **VEGF** | **Leptin** | **PDGF-BB** | **Ang-2** | **Follistatin** | **IL-8** | **Ang-2/VEGF** |
| --- | --- | --- | --- | --- | --- | --- | --- | --- | --- | --- | --- |
| **Serum** |  |  |  |  |  |  |  |  |  |  |  |
| Clinical | Responder | 32,0 | 2752,5 | 1041,9 | 48,3 | 1673,3 | 1338,0 | 810,0 | 165,9 | 10,9 | 20,9 |
|  | Nonresponder | 35,3 | 3398,2 | 1045,4 | 34,8 | 1468,8 | 1192,9 | 968,5 | 214,8 | 12,5 | 22,4 |
| TRG | Responder | 33,9 | 2841,5 | 950,7 | 57,1 | 1456,9 | 1500,6 | 1002,4 | 453,3 | 13,7 | 22,2 |
|  | Nonresponder | 35,7 | 3329,6 | 1045,4 | 34,8 | 1636,9 | 1180,4 | 927,6 | 209,0 | 12,3 | 22,4 |
| **Tissue** |  |  |  |  |  |  |  |  |  |  |  |
| Clinical | Responder | 5,7 | 38461,9 | 4848,4 | 41,8 | 100,1 | 33,3 | 396,4 | 506,7 | 179,7 | 10,3 |
|  | Nonresponder | 6,1 | 26760,9 | 5394,0 | 39,2 | 108,2 | 35,6 | 316,1 | 408,5 | 59,7 | 4,3 |
| TRG | Responder | 6,1 | 34933,9 | 4848,4 | 24,9 | 94,7 | 28,7 | 316,1 | 408,5 | 37,5 | 10,3 |
|  | Nonresponder | 6,1 | 26901,4 | 4923,5 | 48,8 | 113,6 | 36,2 | 333,2 | 398,6 | 64,4 | 4,2 |
| **SCC** |  | **G-CSF** | **PECAM-1** | **HGF** | **VEGF** | **Leptin** | **PDGF-BB** | **Ang-2** | **Follistatin** | **IL-8** | **Ang-2/VEGF** |
| **Serum** |  |  |  |  |  |  |  |  |  |  |  |
| Clinical | Responder | 28,3 | 3260,8 | 943,4 | 43,5 | 1916,7 | 1698,5 | 1003,6 | 225,4 | 12,3 | 23,4 |
|  | Nonresponder | 38,5 | 2699,2 | 907,2 | 36,8 | 1079,8 | 1299,5 | 697,0 | 190,8 | 11,0 | 18,2 |
| TRG | Responder | 34,8 | 3121,9 | 907,2 | 37,4 | 1898,3 | 1466,2 | 687,7 | 188,8 | 10,9 | 16,3 |
|  | Nonresponder | 45,5 | 2600,4 | 1002,0 | 35,6 | 993,2 | 1317,1 | 842,5 | 218,7 | 11,3 | 22,4 |
| **Tissue** |  |  |  |  |  |  |  |  |  |  |  |
| Clinical | Responder | 4,2 | 31955,6 | 3630,1 | 49,9 | 127,2 | 26,6 | 703,8 | 891,9 | 14,9 | 12,2 |
|  | Nonresponder | 11,5 | 29392,7 | 7168,8 | 88,4 | 97,4 | 37,1 | 412,4 | 820,1 | 216,0 | 10,1 |
| TRG | Responder | 4,2 | 29129,5 | 4640,8 | 42,0 | 105,6 | 26,6 | 402,6 | 914,1 | 18,5 | 12,6 |
|  | Nonresponder | 13,1 | 38150,6 | 7531,1 | 108,1 | 138,2 | 42,9 | 661,9 | 772,0 | 509,3 | 10,1 |

**Values are the median values of the particular subgroups in pg/ml; Ang2/VEGF: ratio between Angiopoietin-2 and VEGF**
