# Supplementary material for: Association of angiogenic factors with prognosis in esophageal cancer
Source: BMC Cancer. 2015 Mar 13;15:121. doi: 10.1186/s12885-015-1120-5 (PMC4362831; doi:10.1186/s12885-015-1120-5)
Supplement: Additional file 4: Table S4. — A) Prognostic significance of circulating angiogenic factor levels in AEG I/II. B) Prognostic significance of tissue angiogenic factor levels in AEG I/II. C) Prognostic significance of circulating angiogenic factor levels in SCC. D) Prognostic significance of tissue angiogenic factor levels in SCC. [file 12885_2015_1120_MOESM4_ESM.docx]

**Additional file 4: Table S4:**

**A) Prognostic significance of circulating angiogenic factor levels in AEG I/II**

| **Cytokine Serum AEG I/II** | **Median** |  | **n** | **%** | **Median Survival**  **(IQR)** | **95% CI** | **3-Y-S (%)** | **p value** |
| --- | --- | --- | --- | --- | --- | --- | --- | --- |
| G-CSF | 35,7 | ≤ Median | 26 | 60,5% | 28,5 (17;*) | 17,6 - 39,4 | 37,7% | 0,739 |
|  |  | > Median | 17 | 39,5% | 30,6 (21,1;*) | 18,9 - 42,3 | 39,6% |  |
| PECAM-1 | 3295,3 | ≤ Median | 22 | 51,2% | 29,1 (13,8;*) | 17,9 - 40,3 | 42,7% | 0,621 |
|  |  | > Median | 21 | 48,8% | 28,5 (20,3;*) | 13,3 - 43,7 | 33,0% |  |
| HGF | 1023,4 | ≤ Median | 22 | 51,2% | 23,8 (17;*) | 11,6 - 36,0 | 38,1% | 0,780 |
|  |  | > Median | 21 | 48,8% | 29,1 (20,3;*) | 16,5 - 41,7 | 38,8% |  |
| VEGF | 37,1 | < Median | 21 | 48,8% | 23,8 (12,8;*) | 13,9 - 33,7 | 34,5% | 0,491 |
|  |  | > Median | 21 | 48,8% | 33,7 (19,1;*) | 16,9 - 50,5 | 41,5% |  |
| Leptin | 1624,9 | < Median | 21 | 48,8% | 30,6 (20,3;*) | 15,6 - 45,6 | 41,9% | 0,592 |
|  |  | > Median | 21 | 48,8% | 28,5 (13,8;*) | 18,7 - 38,3 | 34,7% |  |
| PDGF-BB | 1185,4 | ≤ Median | 22 | 51,2% | 24 (13,7;*) | 15,1 - 32,9 | 35,1% | 0,521 |
|  |  | > Median | 21 | 48,8% | 33,7 (19,1;*) | 15,1 - 52,3 | 41,5% |  |
| Angiopoietin-2 | 941,6 | ≤ Median | 22 | 51,2% | 24,0 (19,1;*) | 14,3 - 33,7 | 35,2% | 0,558 |
|  |  | > Median | 21 | 48,8% | 33,7 (17,0;*) | 16,6 - 50,8 | 41,1% |  |
| Follistatin | 213,9 | ≤ Median | 22 | 51,2% | 29,1 (21,1;*) | 18,5 - 39,7 | 40,6% | 0,839 |
|  |  | > Median | 21 | 48,8% | 24,0 (13,8;*) | 7,8 - 40,2 | 35,9% |  |
| IL-8 | 12,3 | ≤ Median | 22 | 51,2% | 30,6 (19,1;*) | 23,2 - 38,0 | 40,7% | 0,775 |
|  |  | > Median | 21 | 48,8% | 23,8 (13,8;*) | 19,4 - 28,2 | 35,9% |  |
| Ang-2/VEGF-Ratio | 22,3 | < Median | 21 | 48,8% | 28,5 (19,1;*) | 13,0 - 44,0 | 32,4% | 0,751 |
|  |  | > Median | 21 | 48,8% | 29,1 (13,7;*) | 18,1 - 40,1 | 43,4% |  |

**Median survival shown in months; n.r.: not reached; CI: confidence interval; 3-Y-S: 3-Year-Survival; IQR: inter quartile range (1^st^ quartile; 3^rd^ quartile)**

**B) Prognostic significance of tissue angiogenic factor levels in AEG I/II**

| **Cytokine tissue AEG I/II** | **Median** |  | **n** | **%** | **Median Survival**  **(IQR)** | **95% CI** | **3-Y-S (%)** | **p value** |
| --- | --- | --- | --- | --- | --- | --- | --- | --- |
| G-CSF | 6,1 | ≤ Median | 20 | 54,1% | 33,7 (20,3;*) | 18,8-48,6 | 39,0% | 0,195 |
|  |  | > Median | 17 | 45,9% | 21,7 (11,9;*) | 11,0-32,3 | 29,4% |  |
| PECAM-1 | 27041,8 | ≤ Median | 19 | 51,4% | n.r. | - | 56,1% | 0,062 |
|  |  | > Median | 18 | 48,6% | 22,6 (13,7;33,7) | 13,6-31,7 | 17,7% |  |
| **HGF** | **4848,4** | **≤ Median** | **19** | **51,4%** | **n.r.** | **-** | **55,9%** | **0,028** |
|  |  | **> Median** | **18** | **48,6%** | **21,7 (13,7;33,7)** | **15,4-28,0** | **14,8%** |  |
| VEGF | 39,2 | ≤ Median | 19 | 51,4% | 29,1 (20,3;*) | 18,9-39,4 | 37,9% | 0,432 |
|  |  | > Median | 18 | 48,6% | 22,6 (11,9;*) | 0,0-45,9 | 30,7% |  |
| **Leptin** | **108,2** | **≤ Median** | **19** | **51,4%** | **36,7 (20,3;*)** | **-** | **49,2%** | **0,034** |
|  |  | **> Median** | **18** | **48,6%** | **21,7 (11,0;30,6)** | **9,3-34,2** | **19,9%** |  |
| PDGF-BB | 33,3 | ≤ Median | 19 | 51,4% | 33,7 (22,6;*) | 23,4-44,0 | 40,4% | 0,128 |
|  |  | > Median | 18 | 48,6% | 20,3 (11,0;*) | 11,4-29,2 | 30,6% |  |
| Angiopoietin-2 | 316,1 | ≤ Median | 19 | 51,4% | 36,7 (20,3;*) | - | 47,6% | 0,200 |
|  |  | > Median | 18 | 48,6% | 23,8 (13,7;33,7) | 8,9-38,7 | 24,2% |  |
| Follistatin | 408,5 | ≤ Median | 19 | 51,4% | 33,7 (12,8;*) | 17,7-49,8 | 38,1% | 0,720 |
|  |  | > Median | 18 | 48,6% | 23,8 (17,0;*) | 19,7-27,9 | 11,6% |  |
| IL-8 | 59,7 | ≤ Median | 19 | 51,4% | 36,7 (19,1;*) | - | 46,7% | 0,133 |
|  |  | > Median | 18 | 48,6% | 22,6 (12,8;33,7) | 13,8-31,4 | 42,2% |  |
| Ang-2/VEGF-Ratio | 4,7 | ≤ Median | 19 | 51,4% | 33,7 (12,8;*) | - | 48,2% | 0,334 |
|  |  | > Median | 18 | 48,6% | 23,8 (17,0;36,7) | 15,3-32,3 | 20,4% |  |

**Median survival shown in months; n.r.: not reached; CI: confidence interval; 3-Y-S: 3-Year-Survival; IQR: inter quartile range (1^st^ quartile; 3^rd^ quartile)**

**C) Prognostic significance of circulating angiogenic factor levels in SCC**

| **Cytokine serum SCC** | **Median** |  | **n** | **%** | **Median Survival**  **(IQR)** | **95% CI** | **3-Y-S (%)** | **p value** |
| --- | --- | --- | --- | --- | --- | --- | --- | --- |
| G-CSF | 36,675 | < Median | 15 | 48,4% | 23,0 (8,3;*) | - | 45,7% | 0,698 |
|  |  | > Median | 15 | 48,4% | n.r. | - | 50,6% |  |
| PECAM-1 | 3017,56 | ≤ Median | 16 | 51,6% | 18,8 (11,6;*) | 5,1 - 32,5 | 43,8% | 0,632 |
|  |  | > Median | 15 | 48,4% | n.r. | - | 57,3% |  |
| HGF | 921,67 | < Median | 15 | 48,4% | 23,0 (11,6;*) | - | 45,7% | 0,904 |
|  |  | > Median | 15 | 48,4% | n.r. | - | 51,3% |  |
| VEGF | 37,11 | < Median | 15 | 48,4% | 23,0 (11,6;*) | - | 46,7% | 0,899 |
|  |  | > Median | 15 | 48,4% | 20,5 (8,3;*) | - | 49,5% |  |
| Leptin | 1079,84 | ≤ Median | 16 | 51,6% | 23,0 (8,3;*) | - | 47,4% | 0,807 |
|  |  | > Median | 15 | 48,4% | n.r. | - | 51,9% |  |
| PDGF-BB | 1334,81 | ≤ Median | 16 | 51,6% | 14,0 (7,3;*) | 6,7 - 21,3 | 36,5% | 0,094 |
|  |  | > Median | 15 | 48,4% | n.r. | - | 65,0% |  |
| Angiopoietin-2 | 776,075 | < Median | 15 | 48,4% | n.r. | - | 63,8% | 0,075 |
|  |  | > Median | 15 | 48,4% | 12,3 (6,0;*) | 0,0 - 25,1 | 33,3% |  |
| Follistatin | 192,68 | ≤ Median | 16 | 51,6% | n.r. | - | 55,0% | 0,35 |
|  |  | > Median | 15 | 48,4% | 20,5 (6,0;*) | 0,0 - 42,1 | 44,0% |  |
| IL-8 | 10,99 | ≤ Median | 16 | 51,6% | n.r. | - | 56,3% | 0,367 |
|  |  | > Median | 15 | 48,4% | 20,5 (7,3;*) | 5,5 - 35,5 | 42,2% |  |
| **Ang-2/VEGF-Ratio** | **20,8** | **< Median** | **15** | **48,4%** | **n.r.** | **-** | **72,2%** | **0,032** |
|  |  | **> Median** | **15** | **48,4%** | **14,0 (7,3;*)** | **4,9 - 23,1** | **26,7%** |  |

**Median survival shown in months; n.r.: not reached; CI: confidence interval; 3-Y-S: 3-Year-Survival; IQR: inter quartile range (1^st^ quartile; 3^rd^ quartile)**

**D) Prognostic significance of tissue angiogenic factor levels in SCC**

| **Cytokine tissue SCC** | **Median** |  | **n** | **%** | **Median Survival**  **(IQR)** | **95% CI** | **3-Y-S (%)** | **p value** |
| --- | --- | --- | --- | --- | --- | --- | --- | --- |
| G-CSF | 8,0 | ≤ Median | 11 | 52,4% | 20,5 (6,0;*) | 8,3-32,6 | 39,0% | 0,936 |
|  |  | > Median | 10 | 47,6% | 12,3 (8,3;*) | 6,6-18,0 | 40,0% |  |
| PECAM-1 | 31757,8 | ≤ Median | 11 | 52,4% | 14,0 (8,3;*) | 10,4-17,7 | 30,7% | 0,494 |
|  |  | > Median | 10 | 47,6% | 20,5 (7,3;*) | - | 50,0% |  |
| HGF | 5618,5 | ≤ Median | 11 | 52,4% | 16,0 (11,6;*) | 10,4-21,7 | 40,4% | 0,718 |
|  |  | > Median | 10 | 47,6% | 10,4 (7,0;*) | 0,0-30,7 | 40,0% |  |
| VEGF | 59,0 | ≤ Median | 11 | 52,4% | 20,5 (11,6;*) | 11,6-29,3 | 38,4% | 0,662 |
|  |  | > Median | 10 | 47,6% | 10,4 (7,3;*) | 4,2-16,6 | 40,0% |  |
| Leptin | 127,2 | ≤ Median | 12 | 57,1% | 14,0 (6,0;20,5) | 7,0-21,0 | 25,0% | 0,309 |
|  |  | > Median | 9 | 42,9% | n.r. | - | 55,6% |  |
| PDGF-BB | 29,1 | ≤ Median | 11 | 52,4% | 16,0 (8,3;*) | 10,5-21,6 | 27,3% | 0,655 |
|  |  | > Median | 10 | 47,6% | 11,6 (7,3;*) | - | 50,0% |  |
| Angiopoietin-2 | 464,7 | ≤ Median | 11 | 52,4% | 14,0 (5,0;*) | 5,6-22,5 | 31,2% | 0,306 |
|  |  | > Median | 10 | 47,6% | 20,5 (10,4;*) | - | 50,0% |  |
| Follistatin | 820,1 | ≤ Median | 11 | 52,4% | 14,0 (8,3;*) | 5,2-22,9 | 34,1% | 0,809 |
|  |  | > Median | 10 | 47,6% | 16,0 (7,3;*) | - | 45,7% |  |
| IL-8 | 59,7 | ≤ Median | 11 | 52,4% | 16,0 (8,3;*) | 9,4-22,7 | 30,7% | 0,696 |
|  |  | > Median | 10 | 47,6% | 12,3 (7,3;*) | - | 50,0% |  |
| Ang-2/VEGF-Ratio | 11,4 | ≤ Median | 11 | 52,4% | 14,0 (8,3;*) | 8,5-16,1 | 45,5% | 0,782 |
|  |  | > Median | 10 | 47,6% | 16,0 (7,3;*) | 5,4-35,5 | 34,3% |  |

**Median survival shown in months; n.r.: not reached; CI: confidence interval; 3-Y-S: 3-Year-Survival; IQR: inter quartile range (1^st^ quartile; 3^rd^ quartile)**
